# Exploring the Influence of Acid-Base Status on Athletic Performance during Simulated Three-Day 400 m Race

**DOI:** 10.3390/nu16131987

**Published:** 2024-06-21

**Authors:** François Chiron, Mégane Erblang, Bora Gulören, Federica Bredariol, Imad Hamri, Damien Leger, Christine Hanon, Eve Tiollier, Claire Thomas

**Affiliations:** 1 Exercise Biology for Performance and Health Laboratory (LBEPS), Univ Evry, IRBA, University Paris Saclay, 91025 Evry, France; megane.erblang@univ-evry.fr (M.E.); gulorenbora@hotmail.fr (B.G.); federica.bredariol@studenti.unimi.it (F.B.); claire.thomas@univ-evry.fr (C.T.); 2 French Athletics Federation (FFA), 33 Avenue Pierre de Coubertin, 75640 Paris CEDEX, France; christinehanon@gmail.com; 3 Institute of Biomedical Research and Epidemiology of Sport (IRMES), Institut National du Sport de l’Expertise et de la Performance (INSEP), 11, Avenue du Tremblay, 75012 Paris, France; imadhamri75@gmail.com; 4 Université Paris Cité, VIFASOM (Vigilance, Fatigue, Sleep and Public Health), ERC 7330, APHP, Hôtel-Dieu, Centre du Sommeil et de la Vigilance, 1 Place Parvis Notre Dame, 75004 Paris, France; damien.leger@aphp.fr; 5 French National Institute for Sport, Expertise and Performance (INSEP), Research Department, Laboratory Sport, Expertise and Performance, 75012 Paris, France; eve.tiollier@insep.fr

**Keywords:** high-intensity exercise, sodium bicarbonate supplementation, nutritional strategy, anaerobic performance

## Abstract

This study aimed to investigate the ability of highly trained athletes to consistently perform at their highest level during a simulated three-day 400 m race and to examine the impact of an alkaline diet associated with chronic consumption of bicarbonate-rich water or placebo on their blood metabolic responses before and after the three races. Twenty-two highly trained athletes, divided into two groups—one with an alkalizing diet and placebo water (PLA) and the other with an alkalizing diet and bicarbonate-rich water (BIC)—performed a 400 m race for three consecutive days. Performance metrics, urine and blood samples assessing acid-base balance, and indirect markers of neuro-muscular fatigue were measured before and after each 400 m race. The evolution of the Potential Renal Acid Load (PRAL) index and urinary pH highlights the combination of an alkalizing diet and bicarbonate-rich hydration, modifying the acid-base state (*p* < 0.05). Athletes in the PLA group replicated the same level of performance during three consecutive daily races without an increase in fatigue-associated markers. Athletes experienced similar levels of metabolic perturbations during the three 400 m races, with improved lactate clearance 20 min after the third race compared to the first two (*p* < 0.05). This optimization of the buffering capacity through ecological alkaline nutrition and hydration allowed athletes in the BIC group to improve their performance during the third 400 m race (*p* < 0.01). This study highlights athletes’ ability to replicate high-level performances over three consecutive days with the same extreme level of metabolic disturbances, and an alkaline diet combined with bicarbonate-rich water consumption appears to enhance performance in a 400 m race.

## 1. Introduction

During international competitions such as the Olympics or World Championships, world-class athletes could compete multiple times in a single day and perform up to seven high-intensity 400 m races in a single competition. As a 400 m race requires maximal engagement, the significant solicitation in the energy demand by contracting skeletal muscles is marked by a pronounced decrease in phosphocreatine concentration [1] and an elevated rate of oxygen consumption, reaching up to 94% of the maximal value [2] associated with significant muscle and blood metabolic perturbations [2]. Athletes’ performance is then positively correlated with tolerance for homeostatic perturbations [3], with success linked to the ability to produce high levels of lactate and withstand significant decreases in muscle and blood pH bicarbonate concentration [2,4,5].

However, blood acid-base balance biomarkers, reflecting the homeostatic perturbations induced by high-intensity (H-I) exercise, return to their baseline levels in less than ninety minutes after exercise cessation [6]. We, therefore, question the ability of highly trained athletes to mobilize once again a high level of energy and performance to complete three consecutive 400 m races during a competition, and consequently, to induce a high level of metabolic perturbations, even with an organism already heavily solicited by a first 400 m race. Indeed, impairments in muscle buffering capacity [7], in lactate transporters contents [8,9] and transport activity [10,11], as well as in muscle oxidative capacity [12,13,14] after H-I exercise performed until exhaustion have been reported and could affect muscle function, which contributes to significant skeletal muscle fatigue accumulation [5]. Thus, athletes’ ability to efficiently stimulate anaerobic glycolysis and aerobic metabolism, as well as to regulate muscle and blood metabolic perturbations, could be compromised during the subsequent races.

In this context, accumulation of hydrogen ions (H^+^) with H-I exercise has been reported to particularly affect ion regulation [5], enzyme activity [14], muscle protein expression involved in lactate/pH regulation [15], and oxidative phosphorylation [16], and it could contribute to limiting performance during a 400 m race [2,3]. Then, counteracting metabolic acidosis through the enhancement of buffering capacity either by training [7] or by chronic exogenous buffering agent supplementation [17] can be a way to modulate acid balance and limit muscle fatigue during H-I exercise [18] and promote the repetition of the highest performance during consecutive 400 m races in competition.

Additionally, an alkaline diet could also present another natural approach to influence acid-base balance [19,20] and improve 400 m race performance [20]. Without taking artificial dietary supplements, which are typically administered in capsule form and may induce gastrointestinal disorders [21], a single instance of acute consumption of natural water rich in HCO_3_^−^ ions with a high bicarbonate content (4368 mg/L) could be an easy and natural way to restore acid-base balance after aerobic exercise and enhance residual strength following an isokinetic test [22]. Recently, our team has reported that chronic consumption of bicarbonate-rich water combined with an alkaline diet has a significant impact on acid-base balance regulation after acute H-I exercise compared to the placebo condition, without significant effects on performance [23].

Considering that an acute 400 m race performed until exhaustion induces significant metabolic perturbations in both muscle and blood, affecting the regulation of acid-base balance and leading to pronounced muscle fatigue, we therefore, investigated the ability of highly trained athletes to consistently perform at their highest level during a simulated three-day 400 m race. We also evaluated the impact of repeated 400 m races on blood metabolic responses. Additionally, we proposed that changes in alkaline status resulting from chronic consumption of bicarbonate-rich water and an alkaline diet could have beneficial effects on metabolic responses, better regulating acid-base balance and facilitating the repetition of H-I exercises.

## 2. Methods and Materials

### 2.1. Experimental Approach to the Problem

**Figure 1:**
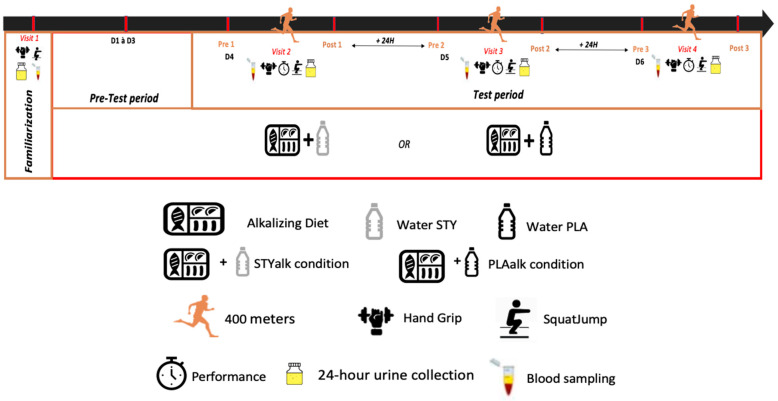
Schematic overview of study design. During the three days leading up to the first 400 m race and throughout the entire protocol, participants followed an alkalizing diet combined with the daily consumption of 2 L of bicarbonate ion-rich water (BIC; n = 11) or placebo water (PLA; n = 11). After Day 4, 5, and 6, the participants engaged in a direct head-to-head simulated competition of 400 m. Various markers and biomarkers were measured before and immediately after each race (post 4′, post 8′, post 20′, and post 1 h).

Participants conducted their first visit (Day 0) during which urine and blood samples were collected. Pre-measurements included a familiarization session involving Handgrip (HG) and squat jumps (SJ), and written consent was obtained. Subsequently, participants were randomly assigned to one of two groups, either an alkalizing diet associated with placebo water (PLA, n = 11) or an alkalizing diet associated with bicarbonate-rich water (BIC, n = 11). Participants were also instructed to modify their habitual diet to adhere to an alkalizing diet (low Potential Renal Acid Load (PRAL) index diet [19]) for the entire 6 days of the experimental period, along with receiving specific dietary recommendations (Figure 1) to achieve this. Throughout the experimental protocol (day 1–day 6), participants were instructed to consume 2 L of either bicarbonate ion-rich water (BIC) or placebo water (PLA) daily, following a double-blind and randomized manner, as part of their daily hydration. In the pre-test period (day 1–day 3), participants were only required to adhere to the dietary and hydration recommendations and maintain a nutritional diary while collecting daily urine samples in a designated container; no other samples were taken. During the final 3 days of the protocol (day 4–day 6), timed tests were conducted for the 400 m race, including pre- and post-race SJ and HG performance assessments, as well as the collection of biomarkers.

### 2.2. Subjects

In accordance with previous studies [20,22], in order to achieve 95% power with a 5% statistical risk, twenty-two (n = 22) highly trained athletes (National Level) from the Athletic French Federation were included in this study (15 males: 22.8 ± 3.3 years, 67.2 ± 7.5 kg, 178 ± 6.9 cm, 400 m race best performance: 51.16 ± 3.54 s and 7 females: 21.3 ± 2.4 years, 58.2 ± 4.4 kg, 170 ± 5.6 cm, 400 m race performance: 59.69 ± 3.56 s). According to the classification by McKay et al., 2022 [24], these highly trained athletes had achieved personal best (PB) performances that met the criteria for Performance level 4 (National 4) as outlined in the Hungarian table of World Athletics Quotation Table (W) for the 400 m race or 800 m race. The Hungarian table is an internationally recognized reference, certified by World Athletics, for classifying athletes based on their performance levels.

All participants were informed of the study protocol, their rights, and the associated risks before providing written informed consent. All procedures were approved by the ethical committee CERSTAPS (CERSTAPS N° 2022-A00644-39/date of approval 15 March 2022) and conducted in accordance with the Declaration of Helsinki (1964, revised 2001).

### 2.3. Procedures

*Experimental water.* During the 6-day protocol, in a double-blind and randomized fashion, participants were instructed to drink 2 L per day of either bicarbonate-rich or placebo water (Table 1). Four bottles of 500 mL each were provided daily to participants (minimum 0.5 L in the morning, 0.5 L in the afternoon, 0.5 L in the hour preceding the 400 m race, and 0.5 L before sleep). St Yorre water (BIC) (Vichy St Yorre, Sources Alma, La Ferrière Bochard, France) is a naturally carbonated water rich in minerals (4774 mg/L containing 4368 mg of HCO_3_^−^ ions). The low-bicarbonate water, or placebo (PLA), was provided to participants in identical bottles to BIC but contained regular carbonated water with considerably lower mineral content (Table 1). Habitual hydration was resumed during the 6 days.

### 2.4. Dietary Intervention

Nutritional suggestions were given to participants during their initial visit. Each participant was given detailed written nutritional guidelines and meal suggestions based on a vegetarian-prone diet [19,25] to follow for the duration of the study. Participants were required to prefer the consumption of vegetables and fruit. Dairy products, including yogurt, fresh cheese, curd, milk, and potatoes were permitted. Participants were asked to avoid consuming meat, fish, and cheese as well as fresh cheese and non-whole wheat starchy foods. The dietary intake was monitored via a written food diary and photographic records of every meal (Guide des portions alimentaires n.d.). The Potential Renal Acid Load (PRAL) was calculated using the equation of Remer and Manz (1995) [19], to characterize their diet.

### 2.5. Urine Analysis

During the familiarization day (Day 0), the three pre-test days (Day 1, Day 2, and Day 3), and each test day (Day 4, Day 5, and Day 6), subjects completed a 24 h urine collection. Urinary pH and urine specific gravity (USG) were assessed using an AL-pH pH meter (Atago Co., Ltd., Saitama, Japan) and a digital pocket refractometer (PAL-1, Atago, Japan).

### 2.6. The 400 m Race Test

During the testing phase (Day 3, Day 4, and Day 5), participants engaged in a daily 400 m race. These races served as simulated competitions, with a specific warm-up, a call room, and a direct confrontation race with a start from starting blocks. For the performance measurements (400 m and 200 m split times (in seconds)), we used video recordings made with an iPhone 11 (1080p HD at 60 frames per second, Apple, Cupertino, CA, USA) and an iPad Pro (1080p HD at 60 frames per second, Apple, Cupertino, CA, USA). A two-handed “clap” was used to start the stopwatch. The video camera was positioned perpendicular to the finish line, similar to official competitions, and the stopwatch was stopped when the athlete’s chest crossed the line. The footage was analyzed using software to obtain measurements accurate to the hundredth of a second. Furthermore, a manual stopwatch was employed to provide a backup measurement in the event of any failure in the primary method. All performance tests were recorded by manual double timing by two athletics coaches, specialists of the French National 400 m team, who took all race times simultaneously to avoid methodological bias.

The 400 m races were conducted on an indoor track with a length of 340 m (Maigrot Hall at the Institute of Sport Expertise and Performance, Paris, France) to ensure highly reproducible conditions (the temperature was maintained at a constant level and there was no wind).

Before each competition, the athletes were encouraged to visualize their perfect race, imagining themselves successfully completing each stage and achieving their goals. They prepared as if for a real competition, using techniques to stay calm and focused. The athletes were conditioned by their coaches similarly to a real competition setting. The training load was adjusted and reduced in the days preceding the tests to ensure they were in optimal shape and motivated to perform. Warming up the athletes lasted one hour as in competition. Athletes were obliged to adhere to their customary competition routine, which included mobilization, jogging, stretching, technical exercises, sprints, and starts, prior to entering the call room. Tactically, the athletes were specifically trained to make a fast and effective start by utilizing their phosphocreatine stores [26] to gain an early advantage. As 400 m specialists, they have also learned to manage their effort with a quick start and to maintain resistance to the inevitable decline in speed that occurs in the final 100 m [2]. Additionally, participants engaged in direct 1 vs. 1 competition with instructions to win the race and achieve the best possible performance, as if they were qualifying for the next round, with their ranking influencing their participation the following day.

Throughout all the tests, the athletes received verbal encouragement and support, similar to a competitive environment, to ensure they felt supported and motivated. Combining these strategies has helped the athletes stay motivated for the three tests and perform at their best in the three 400 m races as they can.

### 2.7. Evaluation of Motivation and Perceived Effort

Immediately after and at 4, 8, 20 min, and 1 h into the recovery phase after the 400 m race, participants were instructed to assess their level of motivation and their perception of effort using the Borg scales [27]. The first scale uses a 7-point scale (1–7) to estimate motivation, where 1 corresponds to “very, very high” motivation and 7 corresponds to ‘very, very low motivation’. The second scale uses a 10-point scale (0–10) to estimate the effort and the perceived exertion of the exercise performed, where a score of 0 corresponds to an exercise that is “very, very easy”, and a score of 10 corresponds to maximal effort.

### 2.8. Handgrip (HG)

During the familiarization session, participants underwent an HG test both before warming up and at four time points: 4 min, 8 min, 20 min, and 1 h after the 400 m race. This test was employed to assess the explosive strength of their forearm muscles, serving as an indirect indicator of central fatigue. Participants were instructed to perform three tests (using the Jamar dynamometer) with a one-minute rest interval between each test. The test was conducted with the dominant arm extended and away from the body. The best performance in kilograms was recorded both before and after each sprint.

### 2.9. Squat Jump (SJ)

During the familiarization session, participants underwent an SJ test both before warming up and at four time points: 4 min, 8 min, 20 min, and 1 h after completing the 400 m race. This test was employed to indirectly assess neuromuscular fatigue relative to the explosive strength of their lower limbs, serving as an indirect indicator of peripheral fatigue. Participants were instructed to perform three squat jumps (using Optojump; Microgate) with a one-minute rest interval between each jump. The test was conducted with hands on their hips, and the knee angle was consistently set at 90 degrees. After stabilizing their position without any backward movement, the experimenter instructed them to jump as high as possible, the best jump performance in centimeters was recorded both before and after each sprint.

### 2.10. Blood Acid-Base Status

Blood biomarkers, including blood bicarbonate concentration ([HCO_3_^−^]), blood pH, blood lactate concentration ([La]), and base excess ([BCef]) were measured via fingertip prick (20 μL) to assess acid-base regulation. Samples were collected during the initial visit (familiarization: Day 0) at rest and on each testing day (Day 4, Day 5, and Day 6) before warm-up (Pre) and after 4 min of exercise (+4′), 8 min (+8′), 20 min (+20′), and 1 h (+1 h) during passive recovery following the 400 m race. The samples were analyzed using a blood gas and electrolyte analyzer (i-STAT, Abbott, Chicago, IL, USA) and a Lactate Pro 2 device (Arkray, LT-1730, Kyoto, Japan).

### 2.11. Statistical Analyses

All values are reported as means ± standard deviations. The Shapiro–Wilk test was used to identify departures from the normal distribution. Performance times, blood gases, lactate values, grip strength (HG), and jump height (SJ) between the first and third 400 m race, as well as between pre-exercise and post-exercise values, were analyzed with a two-way analysis of variance (effects: BIC and PLA). Tukey’s post hoc analysis was undertaken to localize the difference. A priori power analysis indicated that 11 participants per condition were needed to detect significant differences based on an estimated alpha level of 0.05 and a power of 95% based on the exercise performance improvement results from a previous study by Limmer et al., (2018) [20]. The alpha level was set at *p* ≤ 0.05 and was conducted using SPSS 24 (IBM Corp, Endicott, NY, USA). Finally, Cohen’s d scores (average residual/SD) were calculated for performance parameters to categorize the magnitude of the error of estimation as large (0.8), moderate (0.5–0.8), or small (0.5). The Shapiro–Wilk test and Tukey’s post hoc analysis were performed using R (version 3.6.1; The R Foundation for Statistical Computing, Vienna, Austria). All figures were realized with GraphPad Prism software (version 9.1.1 223; extract from https://www.graphpad.com).

## 3. Results 

### 3.1. Food Consumption

The participants consumed an average daily calorie intake of 2457 ± 580 kcal·day^−1^, consisting of 66% carbohydrates, 18% proteins, and 16% fat. The mean PRAL values for BIC and PLA were −5.56 ± 23.22 mEq and −3.09 ± 19.55 mEq, respectively. There was no significant difference in PRAL between the PLA and BIC groups (*p* > 0.05).

### 3.2. Performance

During the experimental phase, the athletes achieved an average of 98% of their personal season’s best performance (PB) in the 400 m race, with a mere difference of a few tenths of a second between their best and their performance in the 400 m race. The performance levels of athletes in the 400 m race between the PLA and BIC groups were not significantly different (*p* > 0.05).

**Figure 2:**
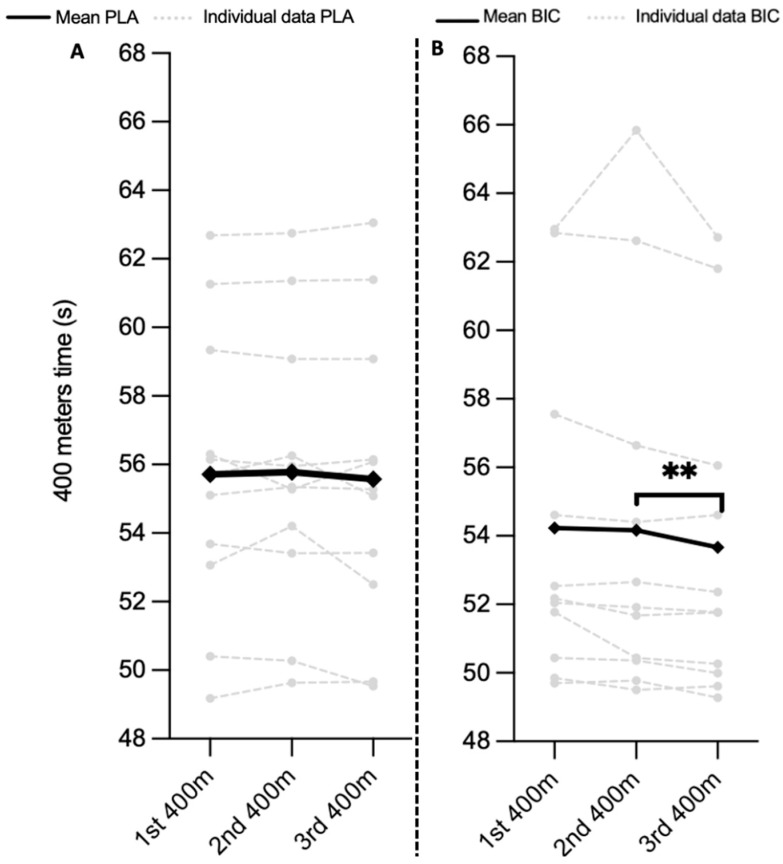
Performance (in seconds). The PLA and BIC group completed a 400 m race on Day 4 (D4), Day 5 (D5), and Day 6 (D6). Significant differences represented by ** *p* < 0.01. (**A**) Tendances de la performance sur 400 m pour le groupe PLA. (**B**) Tendances de la performance sur 400 m pour le groupe BIC.

The PLA group’s average performance after the first 400 m race was 53.70 ± 2.68 s for male athletes and 61.10 ± 1.68 s for female athletes. The average performance after the third 400 m race was 53.47 ± 2.69 s for male athletes and 61.17 ± 1.99 s for female athletes. There was no significant difference between three consecutive 400 m races (*p* > 0.05). The split time at the 200 m race mark for the PLA group between three consecutive daily 400 m races was not significantly different (*p* > 0.05). The time for the last 200 m for the PLA group between the first, the second, and the third consecutive daily 400 m race were not significantly different (*p* > 0.05). (Figure 2A).

The BIC group’s average performance after the first 400 m race was 52.30 ± 2.49 s for male athletes and 62.90 ± 0.70 s for female athletes. The average performance after the third 400 m race was 51.74 ± 1.99 s for male athletes and 62.26 ± 0.65 s for female athletes. The times between the first and second with the third 400 m were significantly different (*p* = 0.01). The intermediate time at 200 m for the BIC group between three consecutive daily 400 m races was not significantly different (*p* > 0.05). The times for the last 200 m for the BIC group between the first and the second with the third 400 m races were significantly different (*p* = 0.01) (Figure 2B). BIC group athletes slowed down less during the second 200 m of the third 400 m race compared to the first (second 200 m of the third 400 m: 28.40 ± 2.75 s vs. second 200 m of the first 400 m: 28.96 ± 2.80 s; *p* = 0.01).

### 3.3. Perception of Effort and Motivation

The motivation and perceived effort between the PLA and BIC groups were not significantly different (*p* < 0.05) during the first and third 400 m races (Table 2).

In the PLA and BIC groups, motivation before the race between the first and third 400 m race was significantly higher (*p* < 0.05). The motivation after the race for the PLA and BIC groups between the first and third 400 m races was not significantly different (*p* > 0.05) (Table 2A,B).

For both the PLA and BIC groups, the perceived effort between the first and third 400 m race was not significantly different (*p* > 0.05) (Table 2A,B).

### 3.4. Indirect Fatigue Markers

For the PLA group, the SJ height was not significantly different between pre-race, which was 35.79 ± 5.79 cm, and post-race, which was 33.53 ± 5.38 cm (*p* = 0.09; ES = 0.1), during the first 400 m race. The SJ was significantly different between pre-race, which was 36.26 ± 5.37 cm, and post-race, which was 33.51 ± 6.12 cm (*p* = 0.05; ES = 0.3), during the third 400 m race. SJ measurements before and after were not significantly different between the first and third 400 m races (Table 2A).

**Figure 3:**
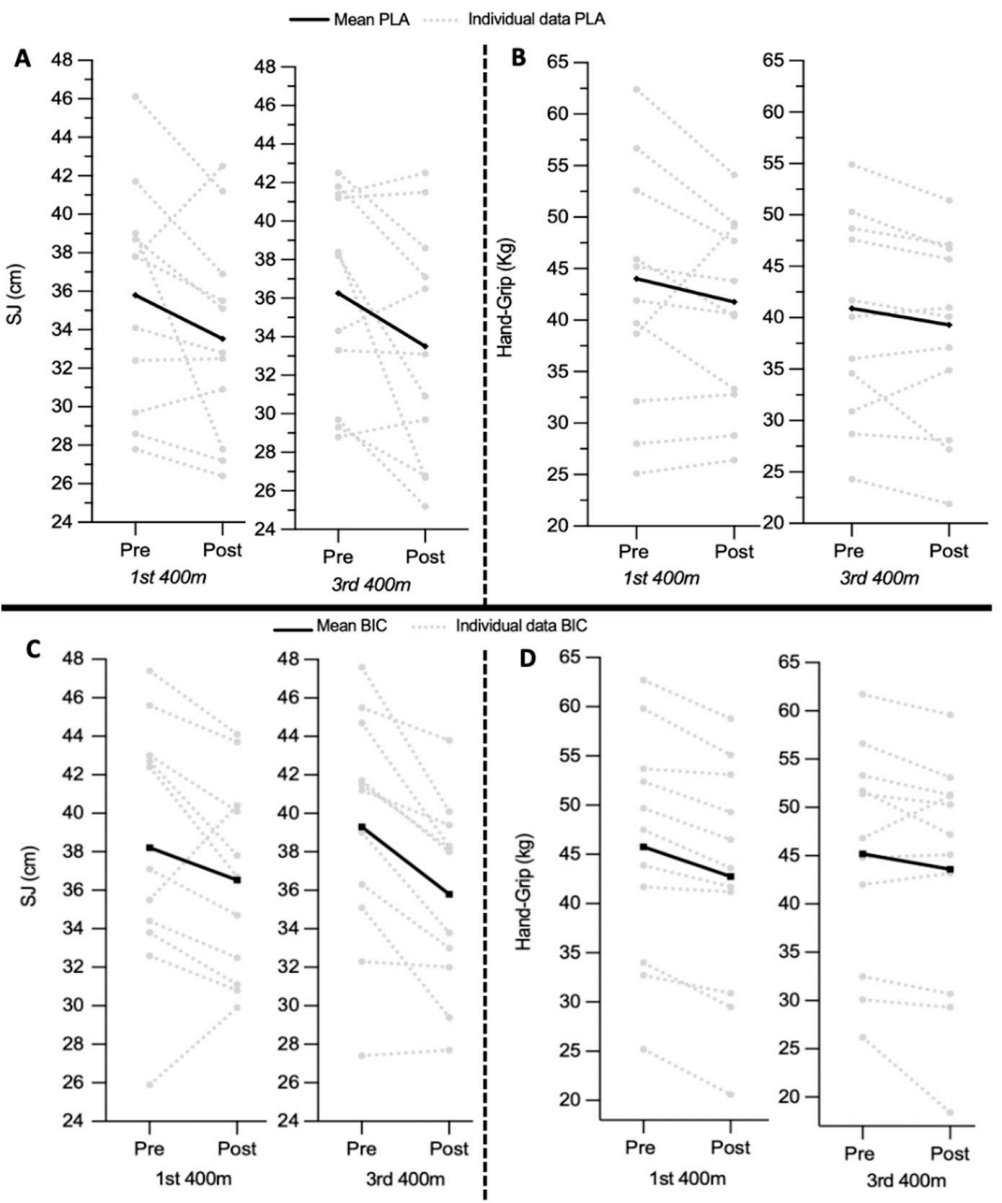
Jump height (SJ in cm) and Handgrip strength (Handgrip in Kg) before and after each 400 m race with passive recovery. Pre = measurement taken before the warm-up; Post 4′ = measurement taken immediately after the 400 m; +8′, +20′, and +1 h = measurements taken 8, 20 min, and 1 h after the 400 m. (**A**,**B**) Jump height (SJ in cm) and Handgrip strength (Handgrip in Kg) before and after each 400 m run with passive recovery for the PLA group. (**C**,**D**) Jump height (SJ in cm) and Handgrip strength (Handgrip in Kg) before and after each 400 m run with passive recovery for the BIC group.

For the BIC group, the SJ height was not significantly different between pre-race, which was 38.22 ± 6.52 cm, and post-race, which was 36.53 ± 5.14 cm (*p* = 0.12; ES = 0.2), during the first 400 m race. However, SJ was significantly different between the pre-race measurement, which was 39.30 ± 6.07 cm, and the post-race measurement, which was 35.79 ± 4.95 cm (*p* < 0.001; ES = 0.4), during the third 400 m race. The SJ measurements before and after the race were not significantly different between the first and third 400 m races (Table 2B). The SJ between the PLA and BIC groups was not significantly different (*p* < 0.05) (Figure 3).

For the PLA group, HG measurements pre-race and post-race were not significantly different during the first and third 400 m races (*p* > 0.05; ES = 0.15). HG measurements pre-race were not significantly different between the first and third 400 m races (Table 2A). In the BIC group, HG measurements between pre-race (before: 45.57 ± 11.67 kg) and post-race (after: 42.75 ± 11.7 kg) during the first 400 m race were significantly different (*p* < 0.001; ES = 0.18). HG measurements between pre-race and post-race during the third 400 m race were not significantly different (*p* = 0.12; ES = 0.13). HG measurements pre-race and post-race were not significantly different between the first and third 400 m races (Table 2B). The HG strength between PLA and BIC groups did not show a significant difference (*p* < 0.05) (Figure 3).

### 3.5. Blood Metabolic Biomarkers

For the PLA group, the minimal average concentrations for the PLA group of bicarbonate [HCO_3_^−^], blood base excess [BCef], and blood pH following the three 400 m race in the PLA group were, respectively, pH: 6.95 ± 0.03; [HCO_3_^−^]: 6.50 ± 1.51 mmol/L; [BCef]: −24.58 ± 2.13 mmol/L. For the PLA group, the concentrations of bicarbonate [HCO_3_^−^], blood base excess [BCef], and blood pH were not significantly different between the first and third 400 m race (*p* > 0.05; as reported in Table 3A).

For the BIC group, the minimal average concentrations of bicarbonate [HCO_3_^−^], blood base excess [BCef], and blood pH following the three 400 m race group were, respectively, pH: 6.95 ± 0.02; [HCO_3_^−^]: 6.67 ± 0.99 mmol/L; [BCef]: −24.65 ± 1,26 mmol/L. For the BIC group, only the pre-concentrations and maximum concentrations of [HCO_3_^−^], blood [BCef], and blood pH were significantly different (*p* < 0.05; as reported in Table 3B).

**Figure 4:**
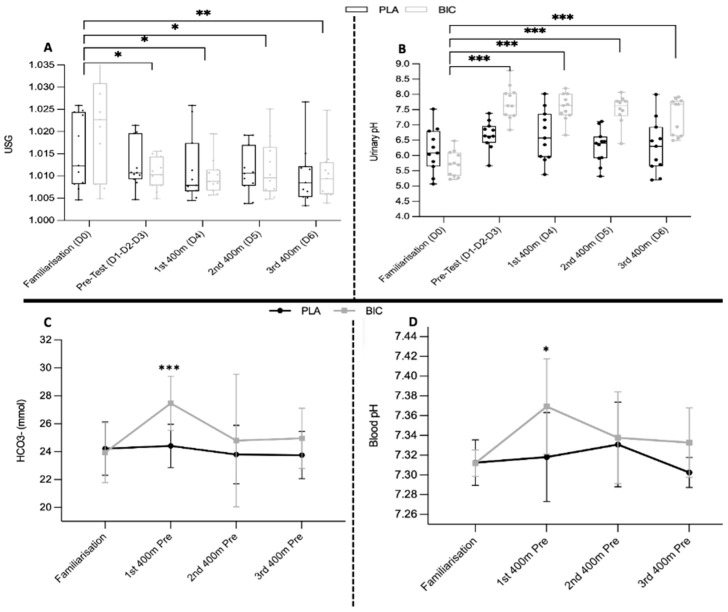
Evolution of biomarkers in urine and blood (**A**,**B**), including urinary pH (pH urinary) and urinary specific gravity (USG). Urine was collected throughout Day 0 (familiarization), D1, D2, and D3 (pre-test) and during the days of simulated competition (D4, D5, and D6). * Indicates a significantly different value (*p* < 0.05) (**C**,**D**). Blood pH and blood bicarbonate concentration ([HCO_3_^−^]) collected on the 1st day (D0 = familiarization) and before warming up during the days of simulated competition (Pre: D4, D5, and D6). Significant differences represented by * *p* < 0.05, ** *p* < 0.01, *** *p* < 0.001.

During the first and third 400 m races for the PLA and BIC groups, the minimum blood [HCO_3_^−^] and pH values occurred at post 8′, while the maximum blood [La] was at post 4′ (Table 3B). The concentrations of bicarbonate [HCO_3_^−^], blood base excess [BCef], and maximum blood pH before and after 1 h for the first and third 400 m were significantly higher for the BIC group compared to the PLA group (*p* < 0.01) (Figure 4C,D). Only the blood [La] at post 1 h during the first and third 400 m race, as well as at post 4′ during the third 400 m race, were significantly higher for the BIC group compared to the PLA group (*p* < 0.03)

The lactate values were significantly higher for the BIC group compared to the PLA group post 1 h with the first 400 m, post 4 min, and post 1 h after the 400 m.

For the PLA group, the maximum blood lactate concentration was 21.33 ± 2.93 mmol/L after the first 400 m and 20.16 ± 1.52 mmol/L after the third 400 m. Only the blood lactate concentration after 20 min was significantly higher between the third and the first 400 m race ([La] first 400 m: 15.99 ± 3.54 mmol/L; [La] third 400 m: 12.92 ± 2.34 mmol/L; *p* < 0.03).

For the BIC group, the maximum blood lactate concentration was 20.46 ± 2.16 mmol/L after the first 400 m and 21.99 ± 2.97 mmol/L after the third 400 m. Only the blood lactate concentration after 20 min was significantly higher between the third and the first 400 m race ([La] first 400 m: 16.48 ± 1.92 mmol/L; [La] third 400 m: 14.26 ± 2.71 mmol/L; *p* < 0.05).

### 3.6. Urinary Markers

Urinary pH measured after a 24 h urine collection was significantly higher (*p* < 0.001) between the first visit (familiarization: D0) and the third, fourth, fifth, and sixth days of BIC water consumption (D3: 7.74 ± 0.55; D4: 7.58 ± 0.44; D5: 7.51 ± 0.48; D6: 7.27 ± 0.60; *p* < 0.001) (Figure 4B). Urinary pH was significantly higher in the BIC group compared to the PLA group after consuming bicarbonate-rich water (BIC) compared to placebo water (PLA) (*p* < 0.001).

Urine specific gravity (USG) measured after a 24 h urine collection was significantly lower (*p* < 0.05) between the first visit (familiarization: D0) and the third, fourth, fifth, and sixth days of BIC water consumption (D0: 1.031 ± 0.003; D3: 1.01 ± 0.001; D4: 1.01 ± 0.001; D5: 1.012 ± 0.001 D6: 1.01 ± 0.001) (Figure 4A). Urine specific gravity did not differ significantly between the BIC and PLA groups after drinking bicarbonate-rich water (BIC) compared to placebo water (PLA) (*p* < 0.001).

## 4. Discussion

Our results suggest that athletes demonstrated the ability to precisely replicate the same level of performance during a simulated three-day 400 m race with no increase in markers associated with fatigue. Athletes were also capable of achieving similarly high levels of metabolic during a competition simulation, involving a sequence of three 400 m races, with an improvement in lactate clearance after a 20 min recovery following the third race compared to the first two. Finally, the positive effects of alkaline nutrition and hydration on the nutritional aspect were also noteworthy, influencing the body’s acid-base status and overall performance.

This study was the first to examine the consequences of a daily sequence of three 400 m races on the physiological determinants of performance. To address this issue, we deliberately recruited highly trained athletes specialized in the 400 m race, training 5 to 6 times per week. During the test period designed to replicate the characteristics of a real competition, this simulated event included a specific warm-up, a call room, a direct confrontation race with a start from starting blocks, and three consecutive races within 72 h, mirroring the format of heats, semi-finals, and finals. Additionally, athletes were required to follow their optimal pacing strategy specifically developed to achieve the best performance in competition [28], which included a fast start and maintaining speed, followed by an inevitable decline. All athletes adhered well to the instructions, achieving 98% of their best season performance, and consistently recorded a second 200 m slower than the first in each 400 m race.

The 400 m race demands a high level of commitment and motivation to achieve optimal performance. The commitment and motivation required for this type of effort are challenging to replicate in laboratory tests, not to mention the specificity of the 400 m race, which involves pace variations, particularly during the final 100 m [2]. Kindermann and Keul (1977) [29] and Schanebel and Kindermann (1983) [30] showed that blood lactate concentrations were significantly higher in high-level athletes during international competitions than after laboratory tests. This is why we chose to conduct these 400 m races in ecological conditions (on the track) rather than in more standardized laboratory conditions. Given the indispensability of this subjective parameter in performance, we assessed the level of motivation and perceived effort difficulty (Borg CR-10) after each 400 m race. These two parameters did not show significant differences between the three races and between conditions of supplementation, indicating a consistent level of athlete engagement throughout the entire protocol (*p* > 0.05; Table 2A,B). Furthermore, a higher level of motivation was observed in both groups prior to the third race in comparison to the first race (*p* < 0.05), as would be expected in a competition with a final.

Continuing the work of Chiron et al., 2024 [23], participants were instructed to follow an alkaline diet, emphasizing the consumption of fruits and vegetables over animal-derived foods combined with the intake of two liters of bicarbonate-rich water or placebo, to influence the body’s acid-base balance. Consistent with Remer and Manz’s (1995) [19] research, the negative PRAL value for the BIC group (−5.56 ± 23.22 mEq) indicated strong adherence to nutritional guidelines. The urinary density in the BIC group was significantly lower than the PLA group from the initial visit until the days following BIC water supplementation, indicating a notable effect. The combination of an alkalizing diet and chronic consumption of bicarbonate-rich water influenced the resting acid-base balance, leading to a significant increase in urinary pH throughout the protocol. This nutritional strategy also impacted blood biomarkers, resulting in elevated blood bicarbonate concentration and blood pH at rest on the third day of the protocol. These changes indicate an enhanced buffering capacity facilitated by both the alkalizing diet and bicarbonate water supplementation.

Furthermore, it should be noted that bicarbonate-rich water contains more sodium and potassium than the placebo, which may improve hydration and potentially anaerobic performance [31]. However, in our study, hydration was not a limiting factor due to the short duration of the event (less than 1 min), similar to findings in endurance performance studies [32]. Additionally, subjects began the exercise well hydrated (USG < 1.02), which did not significantly affect final body temperature or perceived exertion [33]. The additional 220 mg of potassium provided by bicarbonate-rich water was also below the threshold expected to have a significant physiological effect [34,35]. Therefore, the differences in sodium and potassium composition between bicarbonate-rich water and the placebo likely had minimal impact on our results.

The participants in the PLA group were able to achieve three performances at their record level equivalent to 98% of their best season performances in the 400 m race (Table 2). In contrast to the participant levels included in the literature studies, our participants were specialized athletes in the 400 m race, considered expert subjects according to McKay et al. (2022) [24]. The expertise of our participants allowed them to manage their races effectively, thereby avoiding the consequences of pacing strategies on the evolution of blood biomarkers [23]. Furthermore, the large standard deviation (SD) among the subjects could be explained by the fact that the average performances reported after the 400 m races include both male and female athletes. If we consider only the performances of male athletes, the intra-group standard deviation would be, on average, 2.66 s for the PLA group and 1.64 s for the BIC group. This is comparable to the performance variability reported in previous studies [2,3], and it could be assumed that this will have no impact on blood metabolic responses [5,16], which do not differ between sexes from the age of 14 onwards [36].

While the performance of athletes in the PLA group did not show a significant difference, all athletes in the BIC group ran their third 400 m race an average of 0.57 s faster than the first, representing a performance improvement of 1.05% (third 400 m: 53.66 ± 4.74 s compared to first 400 m: 54.23 ± 4.83 s; *p* = 0.01), with no significant changes in blood acid-base balance biomarkers. Specifically, the third 400 m was significantly faster on average for the athletes in the BIC group. It was observed that the BIC group athletes slowed down less during the second 200 m of the third 400 m race compared to the first (second 200 m of the third 400 m: 28.40 ± 2.75 s vs. second 200 m of the first 400 m: 28.96 ± 2.80 s; *p* = 0.01). Therefore, the BIC group exhibited a faster completion time in the second half of the race, corresponding to the final 200 m, during the third 400 m race. Additionally, it is important to note that the subjects served as their own controls, and the study was conducted in a double-blind manner. It could, therefore, be postulated that the observed improvement in performance exhibited by the BIC group between the second and third 400 m races may be attributed to the alkalizing diet in conjunction with bicarbonate-rich water, which may delay the negative effects of metabolic acidosis on muscle performance [23].

In accordance with these results, Chycki et al. (2018) [37] also demonstrated in a double Wingate test involving the lower and upper limbs in trained athletes from combat sports, an increase in power after the consumption of alkaline water versus placebo water. Simultaneously, Limmer et al. (2018) [20] reported also a 2.3% improvement in performance in 400 m races in non-specialized male athletes after implementing an alkalizing diet for 4 days, whereas in our study we found an increase of 1.05% but in high-level athletes who were experts in 400 m race.

Conversely, no performance improvement was demonstrated after an alkalizing diet combined with the consumption of bicarbonate-rich water in a single one-minute Wingate test on a rowing ergocycle [23], which could be explained by the participants’ effort management, leading to various pacing strategies. Furthermore, our present study emphasizes the relevance of implementing nutritional strategies for optimizing performance in the context of H-I exercise sequences. These strategies modify the body’s acid-base balance by optimizing the buffering capacity to improve performance in anaerobic-dominant exercises. Other strategies, such as sodium bicarbonate supplementation through capsules, may optimize the body’s buffering capacity and improve performance [5,33,38]. However, in addition to the fact that some coaches and/or athletes do not wish to systematize this type of supplementation, the implementation of these strategies can also lead to gastrointestinal issues [39]. Thus, the implementation of natural nutritional strategies aimed at modifying the body’s acid-base balance to optimize buffering capacity seems to be effective for improving performance in anaerobic-dominant exercises.

Aiming to anticipate the potential influence of different pacing strategies, we also collected indirect markers of fatigue after each 400 m race using an HG test and an SJ test. In the current study, athletes in the PLA group were able to repeat three consecutive daily 400 m races at 98% of their best season performance without an increase in fatigue, also measured from the grip HG and SJ height. We did not find a significant difference between the pre- and post-levels of central fatigue, evaluated indirectly using an HG [40] after a 400 m race. This result supports those of previous literature, indicating that a 400 m race does not necessarily induce central fatigue [26,41].

Additionally, as SJ performance has often been used as an independent muscular indicator to assess knee extensor fatigue separately from high-intensity sprint exercise [42] and as sprinters exhibited a greater jump difference between performances in the SJ compared to the CMJ [43], we have chosen to use the SJ to indirectly assess neuromuscular fatigue following a 400 m race. The performance in SJ showed an average decrease of 7.5% between the pre-test and post-test, with no significant difference observed during the initial 400 m race (*p* = 0.09; ES = 0.3) and a significant difference noted during the third race (*p* < 0.05). Nummela and colleagues (1992) demonstrated a 39% reduction in jump height between before and after the 400 m race [26]. However, it is worth noting that the exercise employed in this study was a drop jump, which could be considered much more demanding due to the eccentric phase and elastic qualities involved, in comparison to an SJ, which may justify differences with the results obtained in our study [44]. Considering the evolution of these indirect markers of fatigue, we can conclude that in highly trained athletes, three consecutive daily 400 m races do not induce cumulative and delayed fatigue and that pre-exercise metabolic alkalosis has no beneficial effect.

Minimum [HCO_3_^−^] and blood pH values and maximum lactate concentrations during the three consecutive daily 400 m races showed that highly trained athletes were consistently able to activate the glycogenolytic and glycolytic pathways in both PLA and BIC conditions, leading to the exact same level of extreme metabolic disturbances. In PLA and BIC conditions, similar values were observed in the highest blood lactate concentrations after each 400 m race, comparable to those reported in elite and well-trained athletes [2,3]. Furthermore, the lowest blood pH values (first 400 m 6.96 ± 0.04; second 400 m post 4′: 6.98 ± 0.07 mmol; third 400 m post 4′: 6.94 ± 0.04 mmol) and bicarbonate concentrations (first 400 m post 4′: 7.61 ± 0.71; second 400 m, 7.79 ± 1.50 mmol; third 400 m 7.67 ± 1.56 mmol) observed in our study revealed a state of extreme metabolic acidosis, similar to previous results obtained only after an acute simulated 400 m race [2]. This indicates that athletes can produce and sustain extreme blood metabolic perturbations in three days. Subsequently, these metabolic perturbations can be rapidly regulated by athletes due to the development of their physical capacity through training, compared to less-trained or sedentary athletes [45,46,47,48].

Furthermore, the muscular perturbations reported in previous studies on H-I exercise-induced alterations in blood lactate removal [49], lactate transport capacity [11], MCT content [8,9], buffer capacity [7], and oxidative capacity [13,14,16] during and after acute high-intensity exertion performed until exhaustion seem to be transient after each race of recovery. Indeed, contrary to our hypotheses, athletes in this study were able to replicate precisely the same degree of metabolic perturbations every 24 h over three days. This aligns with the notion that training does not protect against the deleterious effects induced by exercise performed until exhaustion on skeletal muscle [7,11]. However, it enables the highest production, transport, and removal of a large quantity of lactate/H^+^, indicating a high energetic demand for glycogenolysis and glycolysis and, therefore, a high ATP production. It also allows for the tolerance of extreme levels of blood metabolic acidosis [4], coupled with a significant depletion of bicarbonate, observed exclusively in elite or world-class athletes [5], and therefore, an improvement in performance.

In addition, a significant decrease in blood lactate concentration was observed in all athletes after a 20 min recovery in PLA and BIC conditions following the third 400 m race (12.92 ± 2.34 mmol.L^−1^ versus 15.99 ± 3.54 mmol.L^−1^ and 16.48 ± 1.92 versus 14.26 ± 2.71 mmol.L^−1^) after the first and third 400 m race. This could be explained by an improvement in blood lactate removal estimated from the mathematical model of Brun et al., 2002 [50], from blood lactate value at 8 and 20 min of recovery, which is in line with previous data in well-trained athletes [47,51,52]. This may indicate an enhancement in lactate transport capacity with or without altering the MCT content [53] and maximal oxidative capacity [54] in these highly trained athletes.

Although we attempted to simulate a competitive context, a championship race represents a considerable psychological stress that can impact athletes’ ability to sustain their performances throughout the competition. Indeed, we observed that the performance level remained consistent for the PLA group, representing an average of 98% of the participants’ performance level. This 2% difference, though not statistically significant, could be crucial at the elite level during a competition, potentially influencing the attainment of an international medal or a place in the final. Therefore, investigating athletes’ capacity in a more ecological context would allow for the assessment of the effects of additional psycho-physiological variables on the ability to replicate a high-level performance.

## 5. Practical Application

Many athletes use sodium bicarbonate supplements to enhance their body’s buffering capacity in sports where the anaerobic lactic energy system plays a significant role. However, taking sodium bicarbonate in capsule form could cause gastrointestinal disturbances [17,21], particularly under the stress of an upcoming international competition, which can impair performance. Additionally, some coaches and athletes may be hesitant to use bicarbonate supplementation in capsule form. As an alternative reported in this study, athletes seeking a more natural and ecological approach can be advised to follow an alkalizing diet (with a negative PRAL value) focused on vegetables and legumes, while limiting animal products, and ensuring a balanced intake of carbohydrates. This should be combined with consuming 1.5 to 2 L of bicarbonate-rich water daily for 3 to 5 days before the event. Based on our previous work [23], we can assert that the nutritional strategy chosen for this study does not cause digestive issues. Nevertheless, to prevent any adverse effects during competition, this should first be tested during training before being applied in a competitive setting.

## 6. Conclusions

In conclusion, athletes specializing in 400 m race events demonstrate the ability to replicate their performance at peak levels for three consecutive days with the same extreme level of metabolic disturbance. The incorporation of an alkaline diet, combined with the consumption of bicarbonate-rich water, appears to enhance the repetition of high-intensity interval exercises during a simulated competition.

## Figures and Tables

**Table 1:** Composition of St Yorre water and placebo water.

Composition of Water	St Yorre (BIC)	Placebo (PLA)
Calcium (mg.L^−1^)	90	93
Magnesium (mg.L^−1^)	11	8.1
Sodium (mg.L^−1^)	1708	8.8
Potassium (mg.L^−1^)	110	2.6
Silica (mg.L^−1^)	16	19
Bicarbonate (mg.L^−1^)	4368	306
Chloride (mg.L^−1^)	322	18
Sulphate (mg.L^−1^)	174	7.5
Nitrates (mg.L^−1^)	2.5	2
Fluorine (mg.L^−1^)	1	0.35
pH	6.6	5.4
Sodium Bicarbonate Equivalent (mg.L^−1^)	6015	-
Sodium Chloride Equivalent (mg.L^−1^)	528	-

**Table 2:** Measurement performance, squat jump, and Handgrip before and motivation and Borg (pre) after (post 4′, post 8′, post 20′, and post 1 h) the 1st and 3rd 400 m for the PLA and BIC groups (N = 11). A difference was considered significant for *p* < 0.05. All data reported as mean ± SD. Significant differences represented by ** *p* < 0.01, *** *p* < 0.001 between pre and post.

**2A**	**PLA**			
**Variables**	**1st 400 m**	**2nd 400 m**	**3rd 400 m**	**1st vs. 3rd 400 m**
	**Mean ± sd**	**Mean ± sd**	**Mean ± sd**	***p* Value**
Performance (s)	55.71 ± 4.19	55.55 ± 4.17	55.57 ± 4.34	0.28
1st 200 m (s)	26.13 ± 1.86	26.22 ± 1.87	26.13 ± 1.86	0.97
2nd 200 m (s)	29.58 ± 2.49 ***	29.33 ± 2.64 ***	29.44 ± 2.57 ***	0.28
Motivation Pre	1.82 ± 0.75	2.27 ± 1.06	2.64 ± 1.12	0.01
Motivation Post	3.00 ± 1.41 **	3.09 ± 1.23 **	3.00 ± 1.10	1
Borg Pre	1.21 ± 1.45	1.18 ± 1.01	1.64 ± 1.16	0.46
Borg Post	4.54 ± 2.73 ***	4.14 ± 2.19 ***	5.00 ± 2.41 ***	0.58
**2B**	**BIC**			
**Variables**	**1st 400 m**	**2nd 400 m**	**3rd 400 m**	**1st vs. 3rd 400 m**
	**Mean ± sd**	**Mean ± sd**	**Mean ± sd**	***p* Value**
Performance (s)	54.23 ± 4.83	54.16 ± 5.45	53.66 ± 4.74	0.01
1st 200 m (s)	25.27 ± 2.09	25.61 ± 2.29	25.40 ± 2.10	0.93
2nd 200 m (s)	28.96 ± 2.80 ***	28.55 ± 3.24 ***	28.40 ± 2.75 ***	0.01
Motivation Pre	1.91 ± 0.83	2.82 ± 1.89	2.64 ± 1.12	0.04
Motivation Post	2.64 ± 1.04	2.82 ± 1.08	2.36 ± 1.03	0.54
Borg Pre	2.59 ± 2.02	2.09 ± 2.22	2.00 ± 1.69	0.13
Borg Post	5.32 ± 1.98 ***	4.77 ** ± 1.79	4.5 ** ± 2.38	0.19

**Table 3:** Measurements of blood bicarbonate concentration, blood pH, blood lactate concentration, and base excess concentration, before (pre) and after (post 4′, post 8′, post 20′, and post 1 h) the 1st, 2nd, and 3rd 400 m for the PLA and BIC groups (N = 11). A difference was considered significant for *p* < 0.05. All data reported as mean ± SD. Significant differences represented by * *p* < 0.05, *** *p* < 0.001; # Significant differences represented between PLA and BIC group.

**3A**	**PLA**			
**Variables**	**1st 400 m**	**2nd 400 m**	**3rd 400 m**	**1st vs. 3rd 400 m**
	**Mean ± sd**	**Mean ± sd**	**Mean ± sd**	***p* Value**
Pre HCO_3_^−^	24.41 ± 1.55	23.79 ± 2.11	23.75 ± 1.69	0.31
Post 4′ HCO_3_^−^	6.70 ± 1.44 ***	7.25 ± 1.36 ***	7.25 ± 1.62 ***	0.27
Post 8′ HCO_3_^−^	6.42 ± 1.63 ***	6.01 ± 1.41 ***	6.58 ± 1.39 ***	0.73
Post 1 h HCO_3_^−^	20.67 ± 2.20 ***	21.72 ± 2.32 *	21.05 ± 2.50 ***	0.48 #
HCO_3_^−^ max	24.41 ± 1.55	23.79 ± 2.11	23.75 ± 1.69	0.31
HCO_3_^−^ min	6.42 ± 1.63 ***	6.01 ± 1.41 ***	6.58 ± 1.39 ***	0.73
pH Pre	7.32 ± 0.05	7.33 ± 0.04	7.30 ± 0.02	0.31
pH Post 4′	6.97 ± 0.05 ***	6.97 ± 0.04 ***	6.97 ± 0.06 ***	0.72
Post 8′ pH	6.95 ± 0.06 ***	6.93 ± 0.05 ***	6.95 ± 0.04 ***	0.33
pH Post 1 h	7.28 ± 0.03	7.31± 0.06	7.29 ± 0.04	0.56
pH min	6.42 ± 1.63 ***	6.01 ± 1.41 ***	6.58 ± 1.39 ***	0.73
pH max	6.95 ± 0.06 ***	6.93 ± 0.05 ***	6.95 ± 0.04 ***	0.33
Pre BCef	−1.20 ± 2.25	−2.18 ± 2.33	−2.64 ± 1.86	0.15
Post 4′ BCef	−24.70 ± 2.06 ***	−24.82 ± 1.94 ***	−24.46 ± 2.21 ***	0.74
Post 8′ BCef	−25.40 ± 2.55 ***	−26.5 ± 2.14 ***	−25.40 ± 1.78 ***	0.85
Post 1 h BCef	−5.65 ± 3.17 ***	−4.45 ± 2.92 *	−5.64 ± 2.80 ***	0.80
BCef min	−25.40 ± 2.55 ***	−26.5 ± 2.14 ***	−25.40 ± 1.78 ***	0.85
BCef max	−1.20 ± 2.25	−2.18 ± 2.33	−2.64 ± 1.86	0.15
Lactate Pre	1.65 ± 0.47	1.74 ± 0.56	1.66 ± 0.36	0.93
Lactate Post 4′	20.49 ± 3.92 ***	19.81 ± 2.40 ***	19.71 ± 1.43 ***	0.46
Lactate Post 8′	19.09 ± 2.68 ***	19.04 ± 0.70 ***	18.75 ± 2.23 ***	0.73
Lactate Post 20′	15.99 ± 3.54 ***	13.93 ± 3.09 ***	12.92 ± 2.34 ***	0.03
Lactate Post 1 h	4.91 ± 1.72 ***	3.77 ± 1.31 ***	4.56 ± 1.35 ***	0.5
Lactate max	20.49 ± 3.92 ***	19.81 ± 2.40 ***	19.71 ± 1.43 ***	0.46
Lactate min	1.65 ± 0.47	1.74 ± 0.56	1.66 ± 0.36	0.93
**3B**	**BIC**			
**Variables**	**1st 400 m**	**2nd 400 m**	**3rd 400 m**	**1st vs. 3rd 400 m**
	**Mean ± sd**	**Mean ± sd**	**Mean ± sd**	***p* Value**
Pre HCO_3_^−^	27.47 ± 1.94 #	24.79 ± 4.75	24.95 ± 2.15	0.001
Post 4′ HCO_3_^−^	7.61 ± 0.71 ***	7.79 ± 1.50 ***	7.67 ± 1.56 ***	0.73
Post 8′ HCO_3_^−^	6.83 ± 0.87 ***	7.05 ± 1.10 ***#	6.51 ± 1.11 ***	0.12
Post 1 h HCO_3_^−^	24.01 ± 2.45 ***#	23.77 ± 2.82	23.26 ± 2.94	0.48
HCO_3_^−^ max	27.47 ± 1.94 #	24.79 ± 4.75	24.95 ± 2.15 #	0.001
HCO_3_^−^ min	6.83 ± 0.87 ***	7.05 ± 1.10 ***	6.51 ± 1.11 ***	0.12
pH Pre	7.37 ± 0.05 #	7.34 ± 0.05	7.33 ± 0.04 #	0.03
pH Post 4′	6.96 ± 0.04 ***	6.98 ± 0.07 ***	6.94 ± 0.04 ***	0.16
Post 8′ pH	6.97 ± 0.06 ***	6.97 ± 0.05 ***#	6.94 ± 0.02 ***	0.18
pH Post 1 h	7.32 ± 0.05 *#	7.31 ± 0.04 *	7.31 ± 0.03	0.56
pH min	6.96 ± 0.04 ***	6.97 ± 0.05 ***	6.94 ± 0.04 ***	0.16
pH max	7.37 ± 0.05 #	7.34 ± 0.05	7.33 ± 0.04	0.03
Pre BCef	2.33 ± 2.74 #	−0.09 ± 3.24	−1 ± 2.41	0.03
Post 4′ BCef	−24.67 ± 0.50 ***	−23.8 ± 2.44 ***	−24.64 ± 2.01 ***	0.63
Post 8′ BCef	−25.13 ± 1.81 ***	−24.8 ± 1.87 ***	−25.89 ± 1.36 ***	0.85
Post 1 h BCef	−2.3 ± 3.09 ***#	−2.45 ± 3.17 ***	−2.91 ± 3.45 #	0.94
BCef min	−25.13 ± 1.81 ***	−24.8 ± 1.87 ***	−25.89 ± 1.36 ***	0.85
BCef max	2.33 ± 2.74	−0.09 ± 3.24	−1 ± 2.41	0.03
Lactate Pre	1.5 ± 0.32	1.75 ± 0.46	1.42 ± 0.36	0.44
Lactate Post 4′	21.69 ± 1.45 ***	21.27 ± 2.17 ***	21.45 ± 2.98 ***#	0.78
Lactate Post 8′	20.31 ± 2.28 ***	20.37 ± 2.38 ***	20.63 ± 2.94 ***	0.75
Lactate Post 20′	16.48 ± 1.92 ***	15.51 ± 2.46 ***	14.26 ± 2.71 ***	0.05
Lactate Post 1 h	3.62 ± 1.14 ***#	3.18 ± 0.80 ***	3.39 ± 1.32 ***#	0.64
Lactate max	21.69 ± 1.45 ***	21.27 ± 2.17 ***	21.45 ± 2.98 ***	0.78
Lactate min	1.5 ± 0.32 #	1.75 ± 0.46	1.42 ± 0.36 #	0.44

## Data Availability

The authors confirm that the data supporting the findings of this study are available within the article.
